# A Tale of Two Patients

**DOI:** 10.4269/ajtmh.23-0185

**Published:** 2023-07-24

**Authors:** Amanjot Kaur

**Affiliations:** Bhubaneswar, India

The first patient, Mr. A, presented to me in Summer 2017 in a village in north India, with complaints of redness, watering, and itching in the affected eye for 3 days. He was harvesting fruit in the field when something fell in his eye from a tree, leading to redness and watering in the affected eye. Being from a rural area where it was more difficult to obtain care, he ignored it. He thought that some dust fell in his eye and that it would improve in few days. When his symptoms worsened, he came to me at an eye camp. The eye camp was a rural clinic that our team set up for 5 days to address various eye issues for people who could not make it into the city or otherwise afford care. We primarily performed screening for common ophthalmic problems.

I was a mere first-year ophthalmology resident at that time, heading a team of two senior optometrists and one paramedic. We were screening about 150 patients per day, and our main aim was to do refraction and dispense appropriate glasses. We also screened for ophthalmic problems such as cataracts, dry eyes, and diabetic retinopathy. Appropriate medicines were advised for such pathologies, and patients requiring surgery or further evaluation were asked to go our tertiary hospital, which was about 100 km from our camp. I had only one slit-lamp and a direct ophthalmoscope with me.

When Mr. A told his history, I also thought that some dust particle might still be lingering in his eye, leading to these symptoms. But as I scanned his eye under the slit-lamp, I could see 10 to 12 worms wriggling around on his conjunctiva. As soon as the light of slit lamp reached them, they wriggled away from light. They were white, translucent, cylindrical, and 1 to 2 mm long. After application of topical anesthesia, I removed the worms from the eye with forceps with great difficulty. I advised a topical broad-spectrum antibiotic, a topical weak steroid, and a topical lubricant. The worms could penetrate deeper, so it was imperative to look carefully. I examined underneath the lid folds but found no evidence of deeper involvement. I put the worms in a sterile bottle, planning on examining them further when I returned to my parent hospital (a tertiary, multispecialty, government hospital/college), but alas the sample was lost in transportation.

Since then, I’ve read as much of the literature as I can. I have seen various case reports written about ophthalmomyiasis, but since I was unable to identify the worm, I could not read specific literature. I often worried that I would never know the full diagnosis in this case, and I would not be able to learn more about it because such cases are rare—a once in a lifetime opportunity.

Five years later, in Summer 2022, I was practicing in a tertiary hospital in a metropolitan city in south India. It was about 1,800 km from the location of first case. I had undergone rigorous training as cornea fellow for the previous 2 years. I was working as a cornea consultant when my colleague, a comprehensive ophthalmologist, called me for help with an interesting case in the Outpatient Department. This patient, Mr. B, was driving a motorcycle without any eye protection when some dust went into his eye. He was going to his home in a village when this incident happened. Just like Mr. A, he had itching, redness, and watering eyes for the previous 2 days. When I examined Mr. B, to my sheer shock, I could see the same worms wriggling in his eye. This time, in addition to anesthetizing the ocular surface with proparacaine eyedrops, I also paralyzed the worms with a topical anticholinergic agent (tropicamide 1%). Unlike previous case, I could remove the worms with ease. I advised the patient to use a topical broad-spectrum antibiotic, a topical weak steroid, a topical lubricant, and a topical antibiotic ointment. The topical broad-spectrum antibiotic was to treat any bacterial infections enabled by the worms presence or trauma from the worm removal. The topical antibiotic ointment was to smother any remaining worms (if present). Closed eyelids with the ointment creates an anaerobic environment that helps suffocate the worms. I advised Mr. B to apply the topical antibiotic ointment at night before sleep. I asked him to return in few days to look for any remanent worms. I could see no subretinal tracks on ophthalmoscope, indicating no deeper involvement in this case also. There was also no involvement of inner sides of eyelids. To ensure I did not repeat my mistakes from previous occasion, I collected the worms and placed them in both a formalin-filled bottle and on a glass slide with cover slip. I packed the sample and sent my personal driver to the nearby tertiary center with a histopathology facility. Because the sample needed no freezing or special equipment, my driver could easily transport the sample. I also called the pathologist beforehand to ensure that he knew to look out for the sample. Happily, the sample was ready and diagnosed in 3 days. Those worms were diagnosed as first-stage larvae of *Oestrus ovis* (the sheep nasal botfly).

When I learned the final identification, I read the literature in detail and was astonished to see the amount of available reading material. I learned that humans are accidental hosts in such cases. I also learned that practicing in a developing country means that these were not the only cases I would see in my lifetime. So far in my career, I have only experienced these two cases, but I have heard from my seniors about cases involving deeper tissues, leading to necrosis of the face and eye socket by different larvae. The progression depends on the species of larvae. Although larvae of *Oestrus ovis* usually do not penetrate deep into tissue, there are a few case reports from our subcontinent describing deeper tissue involvement. Therefore, timely diagnosis and removal becomes imperative. This story reminds us that learning is a lifelong process for a doctor and that we should never lose their curiosity.

